# New Advances in Metastatic Urothelial Cancer: A Narrative Review on Recent Developments and Future Perspectives

**DOI:** 10.3390/ijms25179696

**Published:** 2024-09-07

**Authors:** Elena Tonni, Marco Oltrecolli, Marta Pirola, Cyrielle Tchawa, Sara Roccabruna, Elisa D’Agostino, Rossana Matranga, Claudia Piombino, Stefania Pipitone, Cinzia Baldessari, Francesca Bacchelli, Massimo Dominici, Roberto Sabbatini, Maria Giuseppa Vitale

**Affiliations:** 1 Division of Oncology, Department of Oncology and Hematology, University Hospital of Modena, 41124 Modena, Italy; 325770@studenti.unimore.it (E.T.); 297143@studenti.unimore.it (M.O.); 310546@studenti.unimore.it (M.P.); 308106@studenti.unimore.it (C.T.); 279224@studenti.unimore.it (S.R.); 297407@studenti.unimore.it (E.D.); 297415@studenti.unimore.it (R.M.); 256171@studenti.unimore.it (C.P.); pipitone.stefania@aou.mo.it (S.P.); baldessari.cinzia@aou.mo.it (C.B.); massimo.dominici@unimore.it (M.D.); sabbrob@unimore.it (R.S.); 2 Clinical Trials Office, Division of Oncology, Department of Medical and Surgical Sciences for Children & Adults, University of Modena and Reggio Emilia, 41124 Modena, Italy; francesca.bacchelli@unimore.it; 3 Laboratory of Cellular Therapy, Division of Oncology, Department of Medical and Surgical Sciences for Children & Adults, University of Modena and Reggio Emilia, 41124 Modena, Italy

**Keywords:** antibody–drug conjugate, urothelial cancer, enfortumab vedotin, sacituzumab govitecan, HER2, FGFR, erdafitinib, immune checkpoint inhibitors

## Abstract

The standard of care for advanced or metastatic urothelial carcinoma (mUC) was historically identified with platinum-based chemotherapy. Thanks to the advances in biological and genetic knowledge and technologies, new therapeutic agents have emerged in this setting recently: the immune checkpoint inhibitors and the fibroblast growth factor receptor inhibitors as the target therapy for patients harboring alterations in the fibroblast growth factor receptor (FGFR) pathway. However, chasing a tumor’s tendency to recur and progress, a new class of agents has more recently entered the scene, with promising results. Antibody–drug conjugates (ADCs) are in fact the latest addition, with enfortumab vedotin being the first to receive accelerated approval by the U.S. Food and Drug Administration in December 2019, followed by sacituzumab govitecan. Many other ADCs are still under investigation. ADCs undoubtedly represent the new frontier, with the potential of transforming the management of mUC treatment in the future. Therefore, we reviewed the landscape of mUC treatment options, giving an insight into the molecular basis and mechanisms, and evaluating new therapeutic strategies in the perspective of more and more personalized treatments.

## 1. Introduction

Urothelial cancer (UC) is the ninth worldwide cancer by incidence, with over 614,000 new cases diagnosed in 2022 [1]. UC presents at diagnosis in advanced stages in around 11% of cases [2] but, unfortunately, tumor recurrence or progression represents a much more frequent event, associated with poor prognosis. Historically, the standard of care for advanced or metastatic urothelial carcinoma (mUC) is identified with platinum-based chemotherapy, in particular with the combination of gemcitabine and cisplatin or carboplatin for unfit patients, which proved similar efficacy but with lower toxicity than the methotrexate, vinblastine, adriamycin and cisplatin (MVAC) regimen [3,4].

One major step forward in the metastatic setting was made with the introduction of immune checkpoint inhibitors (ICIs) targeting programmed cell death (PD-1) or its ligand (PD-L1), as second-line or maintenance therapy after a progression-free first-line treatment [5,6,7]. Moreover, in 2017, the U.S. Food and Drug Administration (FDA) granted accelerated approval to atezolizumab and pembrolizumab as a first-line treatment for platinum-ineligible patients [8]. However, though some may have long-term benefit from ICIs treatment, most of patients are destined to eventually progress.

Thanks to the advances in biological and genetic knowledge and technologies, in these last few years, many new strategies have begun to be explored to address this clinical need. In particular, next generation sequencing (NGS) made it possible to identify an mUC population characterized by Fibroblast Growth Factor Receptor (FGFR) pathway alterations and thus to refine a target therapy. Erdafinitib, a pan-FGFR inhibitor, in a phase II trial showed a 40% response rate in patients with the aforementioned genomic alteration, who progressed after a platinum-based chemotherapy regimen, which led to its accelerated approval by the FDA [9].

**Figure 1:**
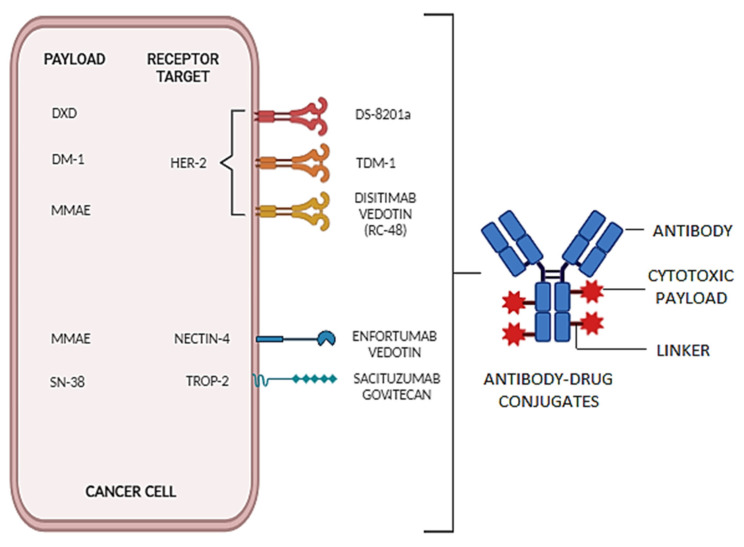
Representation of ADCs structure and the main ones tested for UC. DXD: Deruxtecan, DM-1: Emtansine, MMAE: Monomethyl Auristatin E, HER2: Human Epidermal Growth Factor Receptor 2, T-DM1: Trastuzumab Emtansine, TROP-2: Trophoblast Cell Surface Antigen 2.

**Figure 2:**
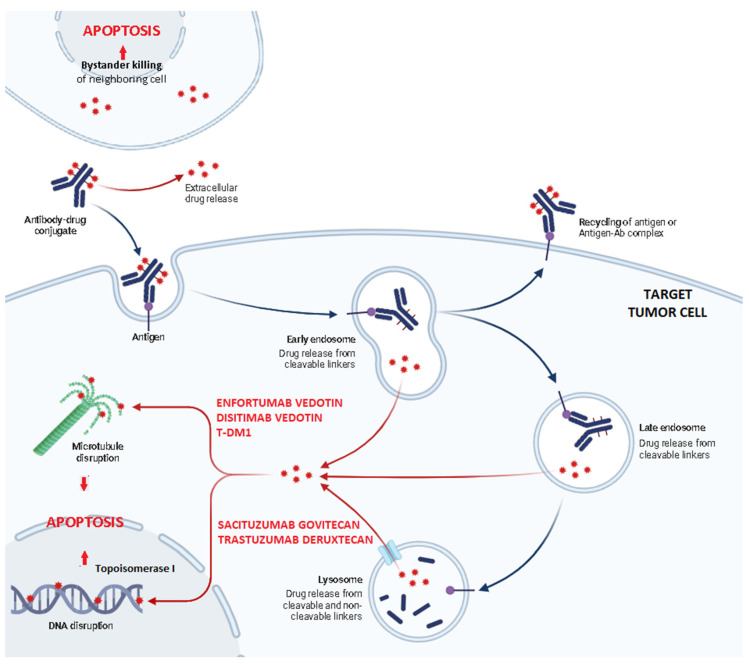
The general mechanism of action of ADCs. ADC molecules circulate in the blood stream until recognizing and binding to their specific antigen. The antigen–ADC complexes are later endocytosed: a few of them are recycled and exposed on the cell membrane, while the majority, thanks to the intracellular environment, pH and lysosomal proteases, end up releasing the cytotoxic agent attached by cleavable or non-cleavable linkers. At this point, the drug is able to induce apoptosis by either interfering with tubulin polymerization and microtubules assembling (EV, DV, TDM-1) or inducing DNA damage, directly or through the inhibition of Topoisomerase I (SG, T-DXd). Some drug molecules are also released in the extracellular space and determine the so-called “bystander killing effect” by inducing apoptosis in neighboring cells too.

More recently, a new class of agents has entered the landscape of mUC treatment: that of antibody–drug conjugates (ADCs) [10,11]. Already known for their use in hematologic malignancies and breast cancer treatment, these agents are formed by three major structural components: a monoclonal antibody (mAb) which recognizes and binds to a specific antigen expressed on cancer cells, a cytotoxic drug (or payload) which is later released into the tumor cell, and a linker molecule which stabilizes the structure while in the blood stream and facilitates drug delivery into the target cell (Figure 1). The mAb binds to an antigen expressed on the surface of the tumor cell and promotes endocytosis to allow the intracellular cytotoxic drug delivery (Figure 2). This allows systemic toxicity to be minimized while optimizing cytotoxic activity on the target [12]. In December 2019, enfortumab–vedotin (EV) was the first ADC to enter the scene with FDA approval for mUC refractory to both ICIs and platinum-based chemotherapy. EV has also proven its efficacy in association with pembrolizumab as a first-line treatment [13], obtaining FDA approval and therefore changing the standard therapy sequence. Sacituzumab–govitecan (SG) has shown significant anticancer activity in heavily pretreated patients and has been granted FDA approval too. Many other ADCs are currently still under investigation [14,15,16].

The aim of this narrative review is to provide an overview of the most recent additions to the landscape of mUC treatment options, giving an insight into the molecular basis and mechanisms, while evaluating new therapeutic strategies in the perspective of more and more personalized treatments.

## 2. Genomic Alterations

The Cancer Genome Atlas (TCGA) project includes the study of 131 high-grade muscle-invasive urothelial bladder carcinomas. TCGA represents the main source of current knowledge on UC genomic alterations, and it reports statistically significant mutations identified in 32 genes [17].

Currently, the most clinically important alterations are those related to the FGFR pathway, since the approval of FGFRi as a target therapy. FGFR1-4 are transmembrane tyrosine kinase receptors consisting of a split intracellular tyrosine kinase domain and three extracellular immunoglobulin-like domains [18]. Fibroblast growth factors bind to these receptors and cause activation of RAS/MEK/ERK and PI3K/AKT signaling pathways [19]. FGFR3 dysregulation via mutation and/or overexpression have been noted in 54% of UCs, more frequently (80%) in non-invasive UCs [20]. *FGFR3* genomic alterations were recently documented to be more frequently detected in the papillary urothelial subtype (65.2%) [21]. *FGFR3-TACC* gene fusions are also important in muscle-invasive bladder cancer (MIBC) and appear more likely to be found in young, non-smoking patients [22]. *FGFR1* aberrations account for another 7–14% of cases, in which subsequent MAPK activation promotes cell multiplication and survival [23]. 

**Figure 3:**
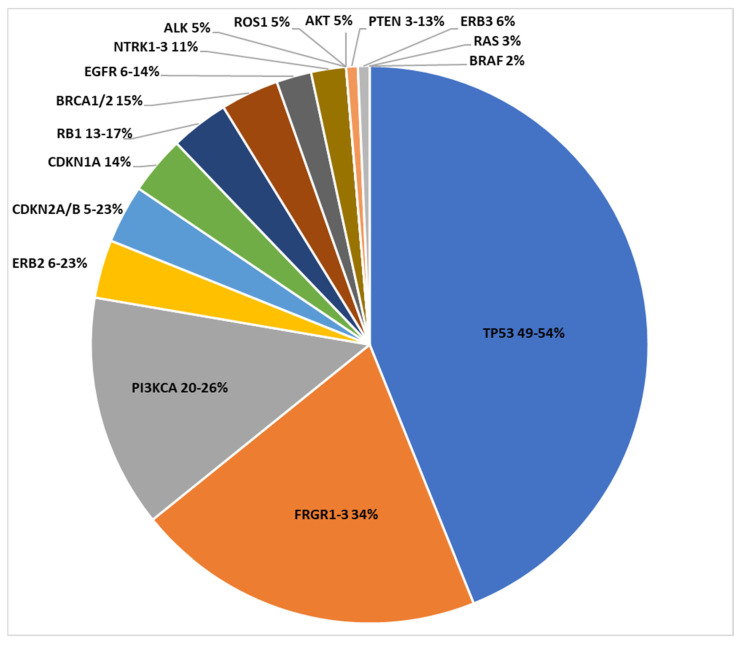
Visual representation of main genomic alterations involved in mUC.

The PI3K/AKT/mTOR pathway, which is involved in cell cycle, differentiation, proliferation and cell movement, is altered in many solid tumors, including bladder cancer, and is associated with poor prognosis [24]. Multiple growth factors, including those belonging to the FGFR and ErbB families, concur to PI3Kactivation. Genetic alterations within this pathway have been reported in 42% of UCs. *PI3KCA*, encoding the catalytic subunit of PI3K, is altered in 20–26% of advanced UCs [25]. *Akt* alterations are detected in 6% of advanced UCs and *PTEN* appears inactivated or deleted in 3–13% of cases (Figure 3) [23]. However, in spite of their biological rationale, inhibitors of the PI3K/Akt/mTOR pathway have proven disappointing results in clinical trials.

In regards to the ErbB family, MIBC has been correlated with mutations or amplifications in *EGFR*, *ErbB-2* and *ErbB-3*. *EGFR* aberrations can be detected in 6–14% of MIBC, *ErbB-2* mutations in 6–23% and *ErbB-3* ones in 6% [12]. *ErbB-2*, in particular, encodes for HER2, a transmembrane tyrosine kinase receptor that promotes cell growth and proliferation. Amplification of *ErbB-2* causes overexpression and hyperactivation of HER2-dependant pathways, therefore stimulating tumour proliferation [26]. Many trials have been and are still evaluating this as a therapeutic target, with promising preliminary results [27,28].

Another pathway involved in UC as in a wide range of malignancies is the MAPK pathway. Growth factors activate the MAPK pathway by binding to their receptors, such as EGFR, resulting in the activation of RAS, which in turn activates RAF and (MEK), which ends up phosphorylating MAPK [29]. Alterations in *RAS* occurs in 2–5% of cases and *BRAF* mutations have also been found in 2% of cases of UCs (Figure 3) [30].

Further, 93% of MIBC cases present with genetic alterations concerning cell cycle regulation. In particular, *CDKN2A/B*, whose function is to inhibit cyclin-dependent kinases CDK4 and CDK6, is altered in 5–23% of MIBC cases; *CDKN1A* and tumour suppressor *RB1* are genetically altered in 14% and 13–17% of cases, respectively. Genetic mutations of *TP53* occur in 49–54% of cases (Figure 3) [17].

Furthermore, genetic alterations in DNA damage response (DDR) have been investigated too. Results show alterations of *BRCA1/2* in 6% and 14% of patients, respectively, and alterations of *ATM* in 12% [25]. Unfortunately, PARP inhibitors have not demonstrated advantageous results in UC treatment. Similarly, although less than 10% of patients in the TCGA cohort had *ALK, ROS1* and *NTRK1/2/3* alterations [31], agents targeting these alterations have not proven efficacy in UC.

Lastly, microsatellite instability is found in 9% of metastatic upper tract UCs (UTUCs). Despite not representing an actual biological target, it may be used as a response predictor to ICIs treatment [32].

## 3. Fibroblast Growth Factor Receptor Inhibitors 

The FGFR family is constituted by four different transmembrane receptors (FGFR1–4) and a fifth receptor, fibroblast growth factor receptor-like 1 (FGFRL1), with no intracellular tyrosine kinase activity; the bond of fibroblast growth factor ligands (FGFs) is what drives their activation [33]. Once activated, FGFR phosphorylates several downstream signaling proteins, including PI3K-AKT, RAS-MAPK and STAT [34,35]. Dysregulation in FGFR signaling, which normally promotes angiogenesis, cell proliferation and tissue regeneration, is associated with several cancers, including UC [36]. Alterations in *FGFR3*, as already mentioned, are the most common in UC [37]. S249C is the most frequent *FGFR3* mutation in bladder UC, while *FGFR3*-*TACC3* is a common fusion formed by tandem duplication on chromosome 4, that leads to the fusion of *FGFR3* with *TACC3* [36]. It is estimated that approximately 20% of cases of advanced and mUC harbors mutations in *FGFR3* with consequently potential therapeutic benefit by FGFR pathway inhibition [38,39].

Given these assumptions, multi-tyrosine kinase inhibitors targeting FGFR alterations have been investigated in patients with mUC [40]. Erdafitinib is an oral pan-FGFR (FGFR1-4) tyrosine kinase inhibitor (TKI), which causes prolonged inhibition of the FGFR pathway due to receptor uptake in intra-cellular lysosomes [41]. A phase II clinical trial (BLC2001) evaluated the antiblastic activity of erdafitinib in 99 patients with unresectable or mUC harboring a prespecified *FGFR3* mutation or *FGFR2/3* fusion, after progression to platinum-based chemotherapy and/or ICIs. The primary endpoint was overall response rate (ORR). Secondary endpoints included progression-free survival (PFS) and overall survival (OS). The ORR was 40%, with 3% complete responses and 37% partial responses. An additional 39% of patients had stable disease, while 18% experienced progressive disease. Median PFS (mPFS) was 5.5 months and median OS (mOS) was 13.8 months. However, treatment-related grade 3 or higher adverse events (TRAEs), manageable by dose adjustments, were recorded in nearly half of the patients, with zero mortality. The most common TRAEs included hyperphosphatemia (77%), stomatitis (58%), diarrhea (50%) and dry mouth (46%). Central serous retinopathy was seen in 21% of patients, 3% of which were grade 3 [9]. Thanks to these promising results, the FDA granted accelerated approval to erdafitinib on 12 April 2019, for patients with *FGFR3* or *FGFR2* genetic alterations who had progressed during or following platinum-based chemotherapy, including progression within 12 months of neoadjuvant or adjuvant platinum-containing chemotherapy. These positive results led to larger confirmatory studies. The phase III THOR trial compared erdafitinib to chemotherapy [42]. The mOS was 12.1 months with erdafitinib vs. 7.8 months with chemotherapy. Secondary end points were mPFS (5.6 months for erdafitinib vs. 2.7 months for chemotherapy) and ORR (46% with erdafitinib vs. 12% with chemotherapy). Moreover, the phase II NORSE trial explored the combination of erdafitinib with immunotherapy, with preliminary promising results [43].

The development of FGFR-targeted therapies is still expanding. Pemigatinib, also known as INCB054828 or Pemazyre^®^, is an orally active, potent small molecule, selective and reversible ATP-competitive inhibitor of FGFR1-3 tyrosine kinases [44]. The phase II clinical trial FIGHT-201 evaluated its efficacy in 260 patients with advanced or mUC who had previously progressed on one or more lines of therapy or were platinum ineligible. Patients received pemigatinib 13.5 mg once daily continuously (CD) or intermittently (ID) until disease progression or unacceptable toxicity. A total of 204 patients with *FGFR3* mutation or fusion were assigned to cohort A (A-CD *n* = 101, A-ID *n* = 103), while patients with other *FGF/FGFR* genetic mutations (44) or FGFR variants of unknown significance (12) were assigned to cohort B. The primary endpoint was ORR in cohort A-CD (17.8%). Secondary endpoints included ORR in cohorts A-ID (23.3%) and B, duration of response (DOR), PFS, OS and safety. In cohorts A-CD/A-ID, the median DOR was 6.2 months, PFS was 4.0 months and OS 6.8 months. However, its activity was limited in cohort B. The most common TRAEs were diarrhea (44.6%) and alopecia, stomatitis and hyperphosphatemia (42.7% each) [45]. An ongoing phase II randomized study (FIGHT-205, NCT04003610) is studying the efficacy of pemigatinib in combination with pembrolizumab vs. the standard of care chemotherapy or immunotherapy in cisplatin ineligible patients.

Rogaratinib is another oral, potent, selective FGFR1–4 inhibitor [36]. The FORT-1 trial phase II/III compared rogaratinib to standard chemotherapy (docetaxel, paclitaxel or vinflunine) in *FGFR1/3* alteratered mUCs. While both treatments achieved similar ORR (rogaratinib 20.7% vs. chemotherapy 19.3%), rogaratinib did not show a significant improvement in OS (mOS of 8.3 vs. 9.8 months for rogaratinib and chemotherapy, respectively) [46].

Infigratinib is another oral agent targeting FGFR signaling (FGFR1-3) [47]. In a study of 67 patients with platinum-refractory mUC harboring activating *FGFR* alterations, infigratinib demonstrated an ORR of 25.4%. Unexpectedly, the drug showed interesting results for UTUC. Among the eight UTUC patients enrolled, one achieved a complete response, while three obtained partial responses. Moreover, the phase III clinical trial PROOF-302 (NCT04197986) is currently investigating infigratinib as an adjuvant therapy for patients with *FGFR3*-mutated MIBC who are either ineligible for or refuse cisplatin-based chemotherapy [48].

## 4. Immunotherapy and Its Synergy with Antibody–Drug Conjugates (ADCs)

UC is an immune-responsive cancer: the first evidence of this was demonstrated by the activity of intravesical Bacillus Calmette–Guerrin (BCG), which by provoking an inflammatory reaction ends up stimulating immune defense against neoplastic cells [49]. Based on this principle, in recent years, ICIs have started to be applied to mUC too. ICIs are immunomodulatory monoclonal antibodies that, by loosening the brake on T lymphocyte activation, enhance the host’s anti-tumor immunity. ICIs bind to PD1 on tumor cells or PDL1 on immune cells, thereby preventing the ligand and receptor from interacting and allowing T cells to activate. 

ICIs have received approval for use in monotherapy in mUC. Pembrolizumab was approved as first-line treatment for platinum-ineligible patients affected by tumors with a PDL1 CPS ≥ 10 according to KEYNOTE-361 trial [50] and as second-line treatment after previous platinum chemotherapy regardless of PD1/PDL1 status according to KEYNOTE-045 [6]. Avelumab was approved as maintenance therapy in patients that did not progress on first-line platinum-containing chemotherapy (JAVELIN Bladder 100 trial) [7]. Lastly, avelumab and nivolumab were granted approval only by the FDA in second line after progression on platinum-based chemotherapy, based on the results of phase Ib and II studies, respectively.

ICIs have also been studied in association with chemotherapy or ADCs. The combination of these agents aims to enhance anti-tumor effectiveness. In fact, cell death, induced by either chemotherapy or ADCs, increases antigen release, leading to an immunogenic tumor microenvironment, thus stimulating immune recognition. The FDA has approved nivolumab in association to gemcitabine and cisplatin in the first-line metastatic setting (CheckMate-901) [51]. However, a combination has not been approved in Europe due to its inferiority when compared with the striking results of the EV-302 trial evaluating the combination of pembrolizumab plus EV [13].

Multiple evidence shows that UC is particularly enriched in antigen expression on its surface and each specific antigen can act as a potential therapeutic target. Many studies addressed several ADCs targeting different antigens, with the common characteristic of delivering cytotoxic drugs to cancer cells with greater therapeutic efficacy and less systemic toxicity [52]. ADCs that have so far shown the most promising results are EV alone (EV-301 trial) [53] or in combination with pembrolizumab (EV-302 trial) [13], SG (TROPHY study) and HER2-targeting ADCs.

## 5. ADCs in mUC

### 5.1. Nectin-4

Enfortumab Vedotin (EV) is an ADC composed of a human anti-Nectin-4 mAb and the antimitotic agent monomethyl auristatin E (MMAE) [54]. Nectin-4 is a cell adhesion molecule that is highly expressed in UC. By binding to Nectin-4, the ADC-Nectin-4 complex is internalized and the intracellular proteases release MMAE, which induces cell cycle arrest and apoptosis by disrupting the microtubule network. Preclinical data prompt that the antitumor activity of EV also includes a bystander killing effect to neighboring cells, suggesting a potential efficacy in cancers with heterogeneous Nectin-4 expression [55] and an immunogenic mechanism that could enhance the action of anti-PD1 agents [56]. According to clinical studies [13,53,57,58,59,60], EV appears as a new promising therapeutic option with a manageable safety profile, that could replace chemotherapy in the management of mUC. It is generally well tolerated and does not require special dose adjustments, even in patients with renal or mild hepatic impairment [61].

Based on results of the phase 3 EV-301 trial (NCT03474107) [53], EV (at a dose of 1.25 mg/kg on days 1, 8 and 15 of each 28-day cycle) is currently recommended for chemo- and immunotherapy-relapsed or refractory mUC. After a median follow-up of almost 24 months, EV confirmed the survival advantages and overlapping toxicity rates compared to chemotherapy, with an mOS of 12.91 vs. 8.94 months (HR 0.704) and an mPFS of 5.55 vs. 3.71 months (HR 0.632), respectively [60]. Furthermore, the ongoing phase 3 EV-302 trial (NCT04223856) showed doubled survival rates with EV (at a dose of 1.25 mg/kg on days 1 and 8 of every 21-day cycle) plus pembrolizumab (at a flat dose of 200 mg on day 1 of every 21-day cycle) compared to chemotherapy in patients with untreated locally advanced or mUC [13]. In December 2023, the FDA granted accelerated approval for EV plus pembrolizumab as a first-line treatment for mUC [62].

Regarding the pre-operative setting, promising results emerged from the EV-103 phase 1b/2 study (NCT03288545). In Cohort L, cisplatin-ineligible pts with untreated MIBC received perioperative treatment with EV (at a dose of 1.25 mg/kg on days 1 and 8 every 21 days) for three neoadjuvant cycles and then for six adjuvant cycles, starting 8 weeks after surgery. In total, 34% and 42% of patients achieved a pathological complete response (pCR) and a pathological downstaging (pDS), respectively [63]. Similarly, in Cohort H, EV was administered in cisplatin-ineligible pts with MIBC for three cycles before radical surgery. At 24 months of follow-up, encouraging data were reported, including a pCR rate of 36.4% (95% CI, 17.2–59.3), a pDS rate of 50.0% (95% CI, 28.2–71.8) and an event-free survival (EFS) rate of 62.0% (95% CI, 38.2–78.9) [64]. Based on this preliminary evidence, phase 3 studies are ongoing to confirm the antitumor activity of EV in MIBC [65,66].

### 5.2. Trophoblast Cell Surface Antigen 2 (Trop-2)

SG is an ADC formed by the hRS7 IgG1k monoclonal antibody conjugated to the topoisomerase I inhibitor SN-38 through a cleavable CL2A linker. The hRS7 IgG1k targets Trop-2, a transmembrane glycoprotein involved in various cellular functions, including proliferation, migration and survival of both stem and tumor cells [67]. Multiple studies confirm high levels of Trop-2 in various cancers like UTUC. This makes Trop-2 an interesting therapeutic target because it is rarely found in healthy tissues. SN-38 is a cytotoxic agent derived from camptothecin; once released into cancer cells, it attacks them by interfering with an enzyme (Topoisomerase-I) thus causing DNA damage and ultimately apoptosis [68,69].

The TROPHY-U-01 trial was a phase II, open-label study designed to evaluate the effectiveness of SG in treating patients with unresectable or mUC [70]. Cohort 1 included patients with either unresectable or mUC who had previously been treated with platinum-based chemotherapy and ICIs, but nevertheless had witnessed disease progression. After a median follow-up of 9.1 months, 27% of patients in the cohort achieved an objective response, and 77% showed a reduction in measurable disease. The mDOR was 7.2 months, while mPFS and mOS were 5.4 months and 10.9 months, respectively. The results of cohort 3 of the TROPHY-U-01 study were promising too. The object of investigation was the combination of SG and pembrolizumab in patients with mUC whose disease had progressed after receiving platinum-based chemotherapy. After a median follow-up of 5.8 months, the ORR observed among the 41 patients enrolled in this cohort was 34% (95% CI, 20.1–50.6; 1 CR; 13 PR), with a median response time of 2.0 months (95% CI, 1.3–2.8). The PFS rate at 6 months was 47% [71]. Cohorts 4 and 5 are yet to be analyzed but will test a combination of SG with cisplatin, either alone or with avelumab as the first-line therapy, followed by avelumab as the maintenance therapy, in patients with mUC who are deemed fit to be administered cisplatin-based chemotherapy [72].

TROPiCS-04 is a currently enrolling, international, phase 3 confirmatory trial. This randomized study is evaluating SG against investigator-selected chemotherapy (vinflunine, paclitaxel or docetaxel) in patients with mUC who have progressed following platinum-based chemotherapy and ICIs (NCT04527991) [73].

### 5.3. Human Epidermal Growth Factor 2 (HER2)

The HER2 positivity rate in mUC varies from 7% to 80%. The higher the UC stage and grade were, the higher HER2 expression was found [74], therefore pointing out its connection to tumor progression and poor prognosis. The enthusiasm coming from the results obtained in breast and gastric cancer has induced many investigators to study the potential of HER2-targeted ADCs in UC too [75]. Here, we analyze ADCs targeting HER2 and in Table 1 we can find all the ongoing clinical trials.

T-DM1 is the first ADC authorized for solid tumor treatment and it is formed by the mAb trastuzumab connected to DM1 by a non-cleavable thioether linker. Trastuzumab is a monoclonal humanized anti-HER2 antibody, while DM1 is the cytotoxic payload, which interrupts the microtubule network formation by binding to tubulin [76]. The phase II study, Kameleon, was meant to study the efficacy of T-DM1 in patients affected by previously treated advanced or metastatic HER2-positive bladder, pancreatic and cholangiocarcinoma cancers. Unfortunately, this study was prematurely closed because of the poor enrolment, but it supported the research of HER2 as a target in UC. In the Kameleon trial, though, T-DM1 showed a partial response in five patients with mUC out of thirteen [77].

Trastuzumab Deruxtecan (T-DXd) is composed of the anti-HER2 mAb trastuzumab connected to the cytotoxic payload, Topoisomerase I inhibitor (TOPO I), through a cleavable tetrapeptide-based linker [76]. DESTINY-Pantumor-02 is an open-label, multi-cohort, multi-center phase II study that showed a benefit in ORR, PFS, DOR and OS thanks to the administration of T-DXd in 267 pre-treated patients affected by different types of HER2-expressing cancers such as bladder, endometrial, cervical, ovarian, biliary tract, pancreatic and other (except for gastric, breast, colorectal and non-small-cell lung cancers). This trial enrolled 41 patients affected by mUC. The ORR was 39% but it was 56.3% in tumors with high HER2 expression (IHC3+). One mUC patient achieved a complete response (2,4%), fifteen had a partial response (36.6%) and sixteen patients showed a stable disease (39%); seven patients, though, were documented with a progression disease (17.1%). Median PFS was 7 months (95% CI: 4.2–9.7 months), while median OS was 12.8 months (95% CI: 11.2–15.1 months). About 85% of all populations treated in this study suffered from a TRAE of any grade, and the most frequent were nausea (55.1%), anemia (27.7%), diarrhea (25.8%), vomiting (24.7%) and fatigue (24.7%). About 41% of all patients underwent a grade 3 or higher drug-related event, and Interstitial Lung Disease (ILD) occurred in 10% of them. Fortunately, it developed as a low grade, but it caused death in three cases [78].

DESTINY-Pantumor-01 is an open-label, phase II basket trial that evaluated the efficacy of T-DXd in patients with unresectable or metastatic solid tumors presenting HER2 as driver mutation. This study enrolled 102 patients who fell into disease progression after previous treatments. The ORR was 29.4% (95% CI: 20.8%–39.3%), while about a half of people suffered from a TRAE of grade 3 or higher. Anemia and neutropenia were the most common, with a frequency of 16% and 8%, respectively. ILD or pneumonitis occurred in 11 patients, mostly low grade, but ILD was fatal for three people [79].

Disitamab vedotin is an ADC made up of three parts: the humanized antibody hertuzumab, a cleavable linker and the cytotoxic payload MMAE. Hertuzumab is more specific for HER2 than trastuzumab and has also shown a greater antibody-dependent cell-mediated cytotoxicity [76].

Two phase II clinical trials, RC48-C005 and RC48-C009, evaluated the efficacy and safety profile of RC48 in 107 Chinese patients affected by HER2-positive (IHC2+ or 3+) mUC, who fell into progression disease after the first line chemotherapy. Combined analysis showed an ORR of 50.5% (95% CI: 40.6%–60.3%). In addition, 31.8% of patients achieved stable disease, 48.6% a partial response, while only 1.9% had a complete response. In this trial, it was also demonstrated that patients with higher HER2 expression (described as either IHC 3+ or HER2 IHC 2+ and FISH-positive) had higher ORR (62.2%), while patients with lower HER2 expression (HER2 IHC2+ and FISH-negative) had lower ORR (39.6%). The overall mOS was 14.2 months (95% CI: 9.7–18.8 months) and the mPFS was 5.8 months (95% CI: 4.2–7.2 months). The most frequent TRAEs were peripheral sensory neuropathy, in more than two thirds of patients (68.2%), leukopenia, in half of them (50.5%), neutropenia (42.1%) and aspartate aminotransferase (AST) elevation (42.1%). Grade 3 TRAEs occurred in 54.2% of the enrolled patients, with peripheral sensory neuropathy and neutropenia being the most common with a frequency of 18.7% and 12.1%, respectively. No grade 4 or 5 TRAEs were reported [80].

RC48-G001 is an ongoing phase 2 study that is testing disitamab vedotin with or without pembrolizumab in treating HER2-positive mUC. Patients are divided into three cohorts according to HER2 expression level and past treatments. Patients in Cohort A and B underwent prior systemic treatments (one- or two-lines therapy), and here disitamab vedotin is administered alone, but the difference between these two groups is the HER2 expression: in Cohort A we can find HER2-positive cancer (IHC2+ or 3+ and FISH-positive) while in Cohort B only HER2-low (IHC 1+ or IHC 2+ and FISH-negative). Cohort C includes patients who did not receive any treatment before, regardless of HER2 expression levels. In this third cohort, patients receive disitamab vedotin both as monotherapy and in combination with pembrolizumab [81].

RC48-C011 is an open-label, single-arm, single centre, phase II trial where disitamab vedotin is administered in HER2-negative mUC patients. Actually, updated data are not available, but this study demonstrated the efficacy and safety of this ADC also in 19 enrolled patients without HER2 expression. Indeed, ORR was 26.3% (95% CI: 9.1–51.2%), mOS was 16.4 months (95% CI: 7.1–21.7 months), while mPFS was 5.6 months (95% CI: 3.9–6.8 months). The TRAEs described were mostly grade 1 and 2 and had similar incidences to those of the RC48-C005 and RC48-C009 trials [82].

## 6. Conclusions

UC is a very heterogeneous disease. Until recently, chemotherapy has represented the only available option for mUC. In these last few years, the mUC treatment landscape has witnessed many advances, starting with the introduction of ICIs, followed by a much more tailored therapy as for erdafitinib for FGFR pathway-mutated patients. ADCs are the last ones to have entered the scene and undoubtedly represent the new frontier, with the potential of changing the standard therapy paradigm in the future. Translational strategies will be crucial to increase the therapeutic index and provide a tailored approach to ADC therapeutic development.

## Figures and Tables

**Table 1:** Clinical trials investigating the aforementioned ADCs: Enfortumab Vedotin, Sacituzumab Govitecan, Trastuzumab Emtansine, Trastuzumab Deruxtecan, Disitimab Vedotin.

ADC	Trial	Drugs	Setting	Phase	Status	Completion Date
**Enfortumab Vedotin**	NCT02091999 [EV-101]	Enfortumab Vedotin	Nectine-4-positive pts, including advanced UC	1	Completed	2022-12-07
	NCT03070990 [EV-102]	Enfortumab Vedotin	Japanese pts with la/mUC	1	Completed	2019-02-25
	NCT03288545 [EV-103]	Enfortumab Vedotin alone and in combination with Pembrolizumab and/or chemotherapy	La/mUC and MIBC	1–2	Active, not recruiting	2026-12-31
	NCT03219333 [EV-201]	Enfortumab Vedotin	La/mUC, previously treated with antiPD1/PDL1	2	Completed	2023-07-28
	NCT03474107 [EV-301]	Enfortumab Vedotin vs. chemotherapy	La/mUC, previously treated with chemotherapy and antiPD1/PDL1	3	Active, not recruiting	2024-08-31
	NCT04223856 [EV-302]	Enfortumab Vedotin +Pembrolizumab vs. chemotherapy	Untreated la/mUC	3	Recruiting	2027-09-30
	NCT03924895 [KN-905/EV-303]	Perioperative Pembrolizumab ± Enfortumab Vedotin	Cisplatin-ineligible or decline cisplatin pts with MIBC	3	Active, not recruiting	2027-12-15
	NCT04700124 [KN-B15/EV-304]	Perioperative Enfortumab Vedotin + Pembrolizumab vs. neoadjuvant chemotherapy	MIBC	3	Active, not recruiting	2026-12-23
**Sacituzumab Govitecan**	NCT05226117	Sacituzumab Govitecan, Preceding Radical Cystectomy	MIBC	2	Unknown status	2023-06 (estimated)
	NCT06133517	PeRioperative Immunotherapy Combined With Sacituzumab Govitecan	MIBC	2	Not yet recruiting	2030-12
	NCT04863885	Ipi/Nivo Plus Sacituzumab Govitecan	Metastatic Cisplatin Ineligible Urothelial Carcinoma	1/2	Active, not recruiting	2025-10
	NCT05535218	Pembrolizumab + Sacituzumab Govitecan	High-risk, Localized Bladder Cancer	2	Enrolling by invitation	2025-09
	NCT05101096 [ASCENT-J02]	Sacituzumab Govitecan	Advanced Solid Tumors	1/2	Recruiting	2026-05
	NCT03992131 [SEASTAR]	Rucaparib and Lucitanib vs. Rucaparib BID + Sacituzumab Govitecan vs. Rucaparib QD and Sacituzumab Govitecan	Advanced/metastatic solid malignancy	1b/2	Terminated	2022-04-22
	NCT05833867	Sacituzumab Govitecan + Adaptive radiotherapy	MIBC	1	Recruiting	2027-10-01
	NCT06161532	Sacituzumab Govitecan ± Atezolizumab	Rare Genitourinary Tumors (SMART) Such as Small Cell, Adenocarcinoma and Squamous Cell Bladder/Urinary Tract Cancer, Renal Medullary Carcinoma and Penile Cancer	2	Not yet recruiting	2028-11-01
	NCT05581589	Sacituzumab Govitecan as Neoadjuvant Therapy	Non-Urothelial Muscle Invasive Bladder Cancer	2	Recruiting	2026-04-30
	NCT04724018	Sacituzumab Govitecan + Enfortumab Vedotin	mUC	1	Active, not recruiting	2026-05-01
	NCT04527991	Sacituzumab Govitecan vs. treatment of physician’s choice (Paclitaxel, Docetaxel or Vinflunine)	Metastatic or Locally Advanced Unresectable Urothelial Cancer	3	Active, not recruiting	2024-10
	NCT05327530	Avelumab vs. Avelumab + Sacituzumab Govitecan vs. Avelumab + M6223 vs. Avelumab + NKTR-255	Maintenance Treatment in Locally Advanced or Metastatic Urothelial Carcinoma with No Disease Progression After First-Line Platinum-Containing Chemotherapy	2	Active, not recruiting	2025-01-23
	NCT03547973 [TROPHY U-01]	Sacituzumab Govitecan	Unresectable Locally Advanced/Metastatic Urothelial Cancer	2	Recruiting	2026-07
	NCT03869190 [MORPHEUS-UC]	Multiple Immunotherapy-Based Treatments and Combinations (Atezolizumab, EV, SG, Niraparib, Magrolimab, Tiragolimab, Tocilizumab, RO7122290)	La/mUC	1b/2	Recruiting	2027-11-27
	NCT04794699	Dose Escalation and Expansion of IDE397 (MAT2A Inhibitor) in monotherapy vs. IDE397 + Paclitaxel/docetaxel vs. IDE397 + Sacituzumab Govitecan	Advanced or metastatic MTAP-deleted advanced solid tumors who are unresponsive to standard of care therapy	1	Recruiting	2027-03-30
**Trastuzumab Emtansine (T-DM1)**	NCT02999672 [KAMELEON trial]	Trastuzumab Emtansine	Bladder cancer, pancreatic cancer, cholangiocellular carcinoma	2	Completed	2018-04-10
	NCT02675829	Ado-Trastuzumab Emtansine	Solid tumor cancer, lung cancer, bladder cancer, urinary tract cancer	2	Recruiting	2025-02
	NCT02465060	Adavosertib; Afatinib; Afatinib Dimaleate; Binimetinib; Capivasertib; Copanlisib; Copanlisib Hydrochloride; Crizotinib; Dabrafenib; Dabrafenib Mesylate; Dasatinib; Defactinib; Defactinib Hydrochloride; Erdafitinib; Fexagratinib; Ipatasertib; Larotrectinib; Larotrectinib Sulfate; Nivolumab; Osimertinib; Palbociclib; Pertuzumab; PI3K-beta Inhibitor GSK2636771; Relatlimab; Sapanisertib; Sunitinib Malate; Taselisib; Trametinib; Trastuzumab; Trastuzumab Emtansine; Ulixertinib; Vismodegib	Solid tumors, lymphomas or multiple myelomas	2	Active, not recruiting	2025-12-31
	NCT04632992	Entrectinib; Inavolisib; Alectinib; Ipatasertib; Atezolizumab; Trastuzumab Emtansine; Pertuzumab, Trastuzumab and Hyaluronidase-zzxf; Tucatinib; Investigator’s Choice of Chemotherapy; Paclitaxel; Tiragolumab; Pralsetinib	Advanced unresectable or metastatic solid tumors	2	Completed	2024-02-27
**Trastuzumab-deruxtecan (T-DXd)**	NCT04482309 [DESTINY-PanTumor02]	Trastuzumab-Deruxtecan	Bladder cancer, endometrial cancer, cervical cancer, ovarian cancer, biliary tract cancer, pancreatic cancer and rare tumors.	2	Recruiting	2027-07-30
	NCT04644068	AZD5305, Paclitaxel, Carboplatin, T-Dxd, Dato-DXd, Camizestrant	Ovarian cancer, breast cancer, pancreatic cancer, prostate cancer, non-small cell lung cancer, small cell, lung cancer, colorectal cancer, bladder cancer, gastric cancer, biliary cancer, cervical cancer, endometrial cancer	2	Recruiting	2026-12-15
	NCT03523572	Trastuzumab-Deruxtecan + Nivolumab	Urothelial and breast cancer	1	Unknown status	2022-07 (estimated)
	NCT04639219 [DESTINY-PanTumor01]	Trastuzumab-Deruxtecan	Unresectable and/or metastatic solid tumors	2	Active, not recruiting	2026-07-14
**Disitamab vedotin (RC48)**	NCT02881190	Disitamab Vedotin	Locally advanced or metastatic solid cancers (gastric, urothelial and others).	1	Completed	2019-11-08
	NCT03507166 [RC48-C005]	Disitamab Vedotin	HER2 overexpressing locally advanced or metastatic UC	2	Completed	2018-10-29
	NCT03809013 [RC40-C009 trial]	Disitamab Vedotin	HER2 overexpressing locally advanced or metastatic UC	2	Completed	2023-06-05
	NCT04879329 [RC48-G001 trial]	Disitamab Vedotin, Pembrolizumab	Urothelial carcinoma	2	Recruiting	2028-05-30
	NCT04073602 [RC48-C011 trial]	Disitamab Vedotin	Locally advanced or metastatic HER2- negative UC	2	Completed	2023-01-31
	NCT05911295	Disitamab Vedotin + Pembrolizumab vs. platinum-based chemotherapy (Cisplatin or Carboplatin) + Gembitabine.	Previously untreated metastatic and locally advanced UC, that express HER2	3	Recruiting	2029-04-30
	NCT05302284	Disitamab Vedotin + Toripalimab vs. platinum-based chemotherapy (Cisplatin or Carboplatin) + Gembitabine.	Previously untreated HER2-expressing unresectable locally advanced or metastatic UC	3	Recruiting	2028-04-30
